# Deep Learning-Based Generation of Retinal Nerve Fibre Layer Thickness Maps from Fundus Photographs: A Comparative Analysis of U-Net Architectures for Accessible Glaucoma Assessment

**DOI:** 10.3390/life16040559

**Published:** 2026-03-29

**Authors:** Kyoung Ohn, Harin Jun, Yong-Sik Kim, Woong-Joo Whang

**Affiliations:** 1 Department of Ophthalmology, Yeouido St. Mary’s Eye Hospital, College of Medicine, The Catholic University of Korea, 10, 63-ro, Yeongdeungpo-gu, Seoul 07345, Republic of Korea; 2 PuzzleAI Co., Ltd., 507 Gangnam-daero, Seocho-gu, Seoul 06536, Republic of Koreapuzzleair@puzzle-ai.com (Y.-S.K.)

**Keywords:** glaucoma, optical coherence tomography, deep learning, retinal nerve fiber layer, U-Net, artificial intelligence

## Abstract

**Introduction:** Optical coherence tomography (OCT) is the gold standard for retinal nerve fibre layer (RNFL) assessment; its high cost and limited accessibility hinder widespread use. This study aims to develop deep learning models that generate RNFL thickness maps from fundus images, providing a cost-effective alternative to OCT. **Methods:** A dataset of 5000 fundus-OCT image pairs from 5000 unique glaucoma patients was used to train and compare the following four U-Net-based deep learning models: ResU-Net, R2U-Net, Nested U-Net, and Dense U-Net. All models were trained for up to 1000 epochs with early stopping (patience = 50 epochs). Performance was evaluated using Mean Squared Error (MSE), Mean Absolute Error (MAE), Peak Signal-to-Noise Ratio (PSNR), Structural Similarity Index Measure (SSIM), and Fréchet Inception Distance (FID). **Results:** ResU-Net demonstrated the best performance, achieving MSE = 0.00061, MAE = 0.01877, SSIM = 0.9163, PSNR = 32.19 dB, and FID = 30.08. These results represent a 108% improvement in SSIM and a 67% improvement in PSNR compared to previously published benchmark for this task. **Conclusions:** This study demonstrates that deep learning models, particularly ResU-Net, can generate high-fidelity RNFL thickness maps from fundus photographs, substantially outperforming prior published benchmarks. This approach represents a potential contribution toward accessible glaucoma assessment, contingent upon prospective clinical validation and regulatory evaluation.

## 1. Introduction

Glaucoma is one of the leading causes of blindness worldwide, characterized by progressive neuropathy with damage to the optic nerve [1]. Early detection and treatment are crucial for preventing vision loss; however, glaucoma is often diagnosed late because it presents with few or nonspecific symptoms in its early stages [2]. Currently, the key imaging technique in glaucoma diagnosis is the analysis of retinal nerve fibre layer (RNFL) thickness using optical coherence tomography (OCT) [3,4]. OCT has established itself as an essential technology for early diagnosis and progression assessment of glaucoma due to its ability to measure RNFL thickness with high precision [5,6,7].

However, OCT equipment is expensive and requires skilled medical personnel, limiting its use in primary care facilities or areas with low medical accessibility. Three specific unmet needs motivate the present study. First, OCT requires specialized equipment, trained operators, and dedicated clinical infrastructure, limiting its utility in point-of-care and primary care settings [5]. Second, in resource-limited and low-income settings, fundus photography is substantially more widely deployed than OCT, as demonstrated by large-scale population-based glaucoma screening programmes [8]. Third, even in settings where OCT is accessible, the additional clinical visit, associated cost, and equipment dependency create practical barriers for glaucoma suspects who do not yet require full diagnostic workup [9]. In contrast, fundus photography is a cost-effective and rapid examination method that can non-invasively capture the structure of the retina and optic disc. Nevertheless, fundus photographs alone cannot directly measure RNFL thickness information. To address this limitation, image transformation technologies utilizing artificial intelligence (AI) are being researched [10].

Recent deep learning and generative AI technologies have established themselves as revolutionary tools in medical image analysis, leading to various ophthalmological imaging studies. Particularly, research on image-to-image translation techniques that predict corresponding images of different types when inputting one kind of medical image has been actively conducted [11]. Previous studies have partially performed image analysis based on Convolutional Neural Networks (CNNs) and OCT image conversion using Cycle-GAN.

Kim et al. [12] enhanced existing OCT image quality using Cycle-GAN but did not generate RNFL structural maps from fundus photographs, limiting direct clinical applicability. Yang et al. [9] predicted regional RNFL thickness values from fundus photographs using a CNN-based approach (sensitivity 92%, specificity 86.9%); however, this method yields scalar thickness estimates rather than full spatial maps, losing the topographic distribution information essential for sector-specific glaucoma assessment. Xu et al. [13] similarly predicted circular RNFL thickness values from fundus photographs without generating spatial maps. Medeiros et al. [14] applied an OCT-trained deep learning algorithm to quantify glaucomatous damage in fundus photographs (Pearson r = 0.832), again without generating full RNFL thickness maps. Chen et al. [10] performed the most directly comparable study, generating RNFL thickness distribution maps from fundus photographs using a style-transfer U-Net; however, their model was trained on only 1120 eyes and achieved SSIM = 0.44 and PSNR = 19.31 dB, indicating limited structural fidelity. Furthermore, none of these studies systematically compared multiple U-Net architectural variants for this specific image generation task.

Generating complete spatial RNFL thickness maps provides clinically essential information beyond what scalar predictions or binary classification can offer. Regression approaches such as those reported by Medeiros et al. [14] and Yang et al. [9] collapse the spatial RNFL distribution into a single global value or a small number of sectoral averages, which is insufficient for identifying focal arcuate defects, assessing superior-inferior sector asymmetry, or correlating RNFL thinning patterns with visual field loss—information critical for glaucoma staging and progression monitoring [15]. Furthermore, our direct image-to-image translation approach using U-Net variants represents a methodological advance over the style-transfer framework of Chen et al. [10] and the numerical prediction models of Yang et al. [9] and Xu et al. [13] by preserving the full two-dimensional topographic structure of the RNFL.

Despite the rapid advancement of generative AI in recent times, there have been few cases actively incorporating it into glaucoma diagnosis and RNFL OCT prediction research. Most studies reported to date have focused on determining the presence of glaucoma using image classification or simple regression models, and there is limited research directly generating RNFL OCT based on fundus photographs using AI.

Several inherent limitations of fundus photography must be acknowledged as constraints on RNFL inference. First, fundus photographs capture only the two-dimensional surface reflectance pattern of the retina and lack the depth-resolved structural information provided by OCT, which measures RNFL thickness with high precision [5,16]. Second, media opacities such as cataract or vitreous hemorrhage degrade fundus image quality and may reduce the reliability of deep learning-based RNFL inference. Third, variability in image quality attributable to pupil dilation status, refractive error, and operator skill introduces heterogeneity in the model input. Fourth, the relationship between fundus reflectance patterns and RNFL thickness is indirect and can be influenced by retinal pigmentation, vascular patterns, and other structural factors [9].

Therefore, the goal of this study is to develop an AI model that generates RNFL OCT maps using fundus photographs as input and to evaluate how similar the predicted OCT is to the actual OCT. To achieve this, we will compare and analyze ResU-Net, R2U-Net, Nested U-Net, and Dense U-Net models utilizing 5000 pairs of fundus photographs and RNFL OCT data. By predicting RNFL thickness using only fundus photographs without OCT, we expect to enable low-cost, high-efficiency early diagnosis of glaucoma that can be utilized even in areas with low medical accessibility. Furthermore, by introducing medical image transformation research based on generative AI, we aim to develop an AI model that generates RNFL thickness maps structurally similar to actual OCT, going beyond simple numerical prediction (regression).

We hypothesized that residual learning-based U-Net architectures would demonstrate superior performance in generating clinically interpretable RNFL thickness maps from fundus photographs compared to dense, nested, and recurrent U-Net variants, as quantified by SSIM [17], PSNR [18], MSE [19], MAE [20], and FID [21] on a held-out test set comprising 500 eyes from patients not included in training or validation.

## 2. Methods

This was a retrospective, single-centre study conducted at Yeouido St. Mary’s Eye Hospital, Seoul, Republic of Korea (IRB No. SC25RISI0075).

**Figure 1:**
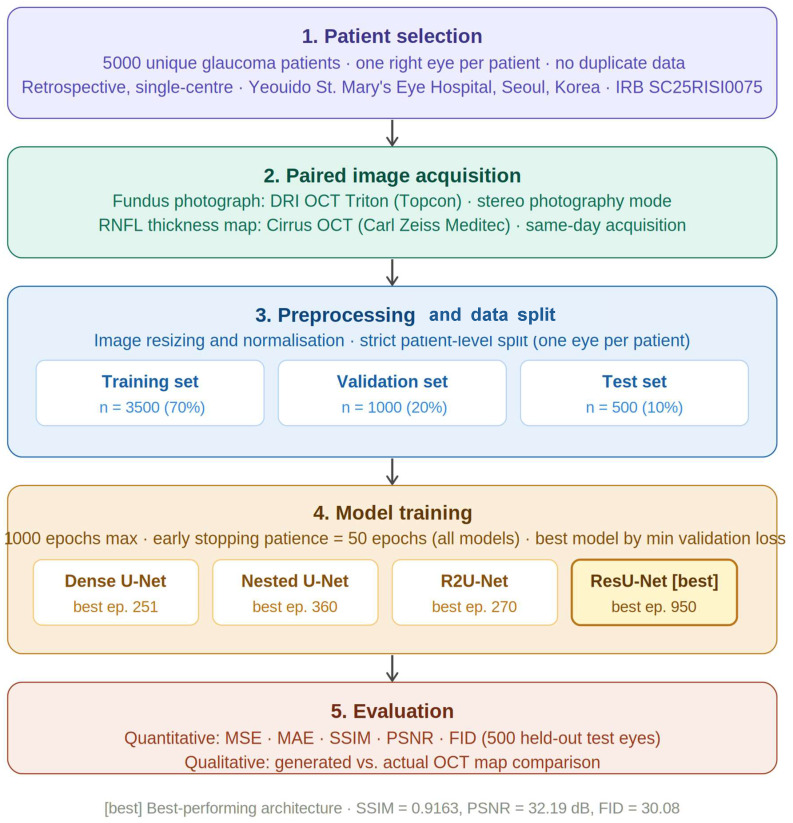
Schematic diagram of the study workflow. Five-stage pipeline: (1) patient selection—5000 unique glaucoma patients, one right eye per patient; (2) paired image acquisition—DRI OCT Triton (fundus) and Cirrus OCT (RNFL map), same-day; (3) preprocessing and patient-level 70/20/10 split; (4) model training—1000 epochs max, early stopping patience = 50 epochs, all models; and (5) evaluation—MSE, MAE, SSIM, PSNR, FID, inference time on 500 test-set eyes. The colors are used for visual clarity and do not indicate any specific categorization.

We retrospectively enrolled all consecutive eligible patients willing to participate, all of whom provided written consent. This retrospective study was approved by the institutional review board of Yeouido St. Mary’s Hospital (IRB No. SC25RISI0075) and was conducted in accordance with the tenets of the Declaration of Helsinki. On the same day, colour fundus photographs were acquired using stereo photography mode of swept-source optical coherence tomography (DRI OCT Triton; Topcon) and retinal nerve fibre layer (RNFL) thickness maps acquired from spectral-domain optical coherence tomography (OCT) with a Cirrus OCT device (Carl Zeiss Meditec). In this study, four deep learning-based models were applied to predict a 1:1 correspondence between fundus photo and OCT-derived RNFL thickness maps. Each model was designed to take fundus photo as input and predict RNFL thickness map, and their performances were compared to identify the optimal prediction model. The overall study workflow is illustrated in Figure 1.

## 3. Data Collection and Preprocessing

Fundus photo and RNFL thickness data from 5000 glaucoma patients were used. A total of 5000 right eyes from 5000 unique glaucoma patients were included; only one eye per patient was used to ensure complete patient-level independence. The 70/20/10 split (training: n = 3500; validation: n = 1000; and test: n = 500) therefore constitutes a strict patient-level partition, with no possibility of the same patient contributing data to more than one subset. The training set was used for model training, the validation set for hyperparameter tuning and model selection, and the test set for evaluating the model’s performance. The fundus images were resized and normalized to be suitable for input into the models, and the fundus images were matched with the corresponding RNFL thickness maps to be used as training data.

Baseline demographic and clinical characteristics of the study cohort are summarized in Table 1. Age, sex, glaucoma subtype, mean deviation on visual field testing, and intraocular pressure are reported.

## 4. Model Architecture

The following four deep learning models were employed to predict RNFL thickness maps: Res U-Net, R2 U-Net, Dense U-Net, and Nested U-Net (UNet++). These models were selected due to their proven efficacy in medical image processing tasks, particularly in extracting detailed structural features from complex datasets. Each model is based on the U-Net architecture, which utilizes an encoder–decoder structure to process OCT images and generate visual field test predictions. The encoder compresses the input image to capture essential features, while the decoder reconstructs the image, focusing on restoring the key visual elements needed for accurate predictions.

Dense U-Net combines the DenseNet and U-Net architectures to maximize information flow between layers [22,23]. Like U-Net, it follows an encoder–decoder structure, but incorporates DenseNet’s key idea, where each layer references all previous layers. This reduces information loss and allows the network to learn more efficiently.

Nested U-Net (UNet++) is an expanded version of U-Net that utilizes multiple skip connections across different layers to minimize information loss [24]. This structure is highly advantageous in learning and restoring complex image features, as it effectively combines features from different resolutions.

R2 U-Net is an extension of the U-Net model that uses recurrent residual connections [25]. This model repeatedly applies the same structure, enabling it to learn deeper and more complex feature representations.

Res U-Net is an improved model that adds residual connections to the U-Net architecture [26,27]. The residual connections mitigate the vanishing gradient problem in deep neural networks, ensuring that the model can maintain learning performance even in deep networks.

## 5. Performance Evaluation Metrics

All models were trained for up to 1000 epochs using the Adam optimizer (learning rate = 2 × 10^−4^, batch size = 4) with MSE loss. Training and validation MSE loss were recorded at every epoch. Early stopping was applied based on validation loss with a patience of 50 epochs for all models. The best-performing model based on minimum validation loss was saved and used for all subsequent evaluations on the test set. Convergence epochs are as follows: Dense U-Net epoch 285, Nested U-Net epoch 360, R2U-Net epoch 270, ResU-Net epoch 950. Batch normalization was applied after convolutional layers to stabilize training.

In this study, five quantitative metrics were used to evaluate the predictive performance of the models. The rationale for selecting each metric and its clinical relevance to RNFL map evaluation are described in detail in Supplementary Materials Section S1. These metrics measure the differences and similarities between generated images and actual images from various aspects.

**(1) MSE (Mean Squared Error):** MSE is a metric used to evaluate the error of the predictive model by calculating the average of the squared differences between the predicted values and the actual values [19].

*MSE* = (1/*N*) × Σ(*x_i_* − *y_i_*)^2^ (*x_i_*, *y_i_*: pixel values of original and predicted images; *N*: total pixels)

**(2) MAE (Mean Absolute Error):** MAE measures the average of the absolute differences between the predicted values and the actual values [20].

*MAE* = (1/*N*) × Σ|*xi* − *yi*|

**(3) SSIM (Structural Similarity Index):** SSIM is a metric that measures the structural similarity between two images [17]. SSIM is designed to mimic the way the human visual system evaluates image quality by considering not only pixel-based differences but also structural information such as luminance, contrast, and texture. SSIM values range from 0 to 1, with higher values indicating greater similarity between the two images.

*SSIM*(*x*,*y*) = (2*μxμy* + *C*1)(2*σxy* + *C*2)/(*μx*^2^ + *μy*^2^ + *C*1)(*σx*^2^ + *σy*^2^ + *C*2)

**(4) PSNR (Peak Signal-to-Noise Ratio):** PSNR is a metric used to measure the difference between two images, often used in evaluating the quality of restored, compressed, or generated images [18]. PSNR is measured in decibels (dB), with higher values signifying smaller differences.

*PSNR* = 10·*log*10(*MAX*^2^/*MSE*)

**(5) FID (Fréchet Inception Distance):** FID measures the difference in distribution between two sets of images, typically used to assess the quality of generated images relative to real images [21]. FID computes the difference between the mean and covariance of two distributions in an embedding space, where smaller values indicate that the generated images are more similar to the real ones.

*FID* = ||*μr* − *μg*||^2^
*+ Tr*(Σ*r* + Σ*g* − 2(Σ*r*Σ*g*)^(1/2))

## 6. Analysis of Optimal Epoch and Predicted Image Quality

All models were trained to convergence (maximum 1000 epochs with early stopping). At every 25 epochs, we evaluated model performance by generating RNFL thickness maps from a single external fundus photograph that was not included in the training set. This approach allowed us to observe prediction quality changes across the four architectures (ResU-Net, R2U-Net, Nested U-Net, and Dense U-Net) as training progressed. Additionally, the changes in the five performance metrics (MSE, MAE, SSIM, PSNR, and FID) were monitored on the test set at every 25 epochs. The epoch with the smallest validation loss across the full training duration was selected as the optimal model, and the generated images as well as the performance on the test set at this epoch were thoroughly examined.

Additional comparisons with prior studies (Table S1) and a summary of metric selection rationale (Table S2) are provided in the Supplementary Materials.

## 7. Results

The performance of four deep learning models (ResU-Net, R2U-Net, Nested U-Net, and Dense U-Net) was evaluated using five quantitative metrics (MSE, MAE, SSIM, PSNR, and FID). Table 2 summarizes the performance metrics of each model at their optimal epoch.

ResU-Net achieved the lowest MSE (0.00061) and MAE (0.01877) among all models, demonstrating excellent pixel-level accuracy in the predicted RNFL thickness maps. R2U-Net showed the second-best performance with MSE of 0.00229 and MAE of 0.03492, followed by Nested U-Net (MSE: 0.00193, MAE: 0.03247). Dense U-Net exhibited the highest error rates with MSE of 0.00380 and MAE of 0.04447, showing significantly poorer performance compared to other models.

The SSIM values showed a similar trend. ResU-Net achieved the highest SSIM value of 0.9163, demonstrating excellent structural preservation of RNFL thickness patterns. R2U-Net and Nested U-Net followed with SSIM values of 0.7475 and 0.7671, respectively. Dense U-Net recorded the lowest SSIM of 0.6621, suggesting substantial structural differences between predicted and actual RNFL maps.

PSNR values further confirmed the superior performance of ResU-Net, which achieved 32.19 dB, while R2U-Net, Nested U-Net, and Dense U-Net recorded 26.45 dB, 27.19 dB, and 24.42 dB, respectively. ResU-Net achieved PSNR = 32.19 dB, exceeding 30 dB and substantially higher than Chen et al. [10] (PSNR = 19.31 dB), indicating reduced reconstruction noise relative to other architectures and prior benchmarks.

FID scores showed that ResU-Net (30.08) produced RNFL thickness maps with distributions most similar to real ones. R2U-Net (81.96) and Nested U-Net (76.18) showed moderate performance, while Dense U-Net showed the poorest performance with an FID score of 62.22.

For contextual benchmarking, Chen et al. [10] reported SSIM = 0.44 and PSNR = 19.31 dB for fundus-to-RNFL map generation using a style-transfer U-Net trained on 1120 eye pairs. The ResU-Net performance in the present study (SSIM = 0.9163, PSNR = 32.19 dB, trained on 5000 eye pairs) represents a 108% improvement in SSIM and a 67% improvement in PSNR relative to this benchmark. However, these metrics reflect image-level structural similarity and should not be equated with clinical diagnostic equivalence to OCT measurements; prospective clinical validation against diagnostic outcomes is required before clinical deployment.

Although ResU-Net achieved the highest structural fidelity among all tested architectures, the achieved metrics represent high but not clinically validated image-level similarity; residual discrepancies between generated and actual RNFL maps may have clinical implications for borderline cases or focal defects, and results should be interpreted accordingly [10,17,18].

The SSIM gap between ResU-Net (0.9163) and the second-best model, Nested U-Net (0.7671), of 0.149 SSIM units represents a difference likely to affect the visual fidelity of structural features—including focal arcuate defects and superior/inferior sector thinning patterns—relevant to glaucoma staging and progression assessment [15]. Formal clinical relevance thresholds for generated RNFL maps have not been established and remain an important area for future validation.

**Figure 2:**
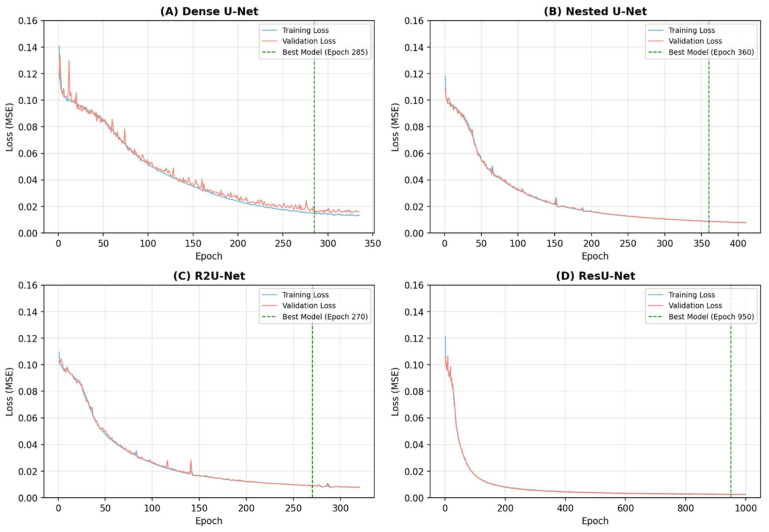
Training and validation loss curves for four U-Net architectures. Training (blue) and validation (red) MSE loss curves shown over full training duration (maximum 1000 epochs; early stopping patience = 50 epochs for all models). Panel (**A**) Dense U-Net: best epoch 285; (**B**) Nested U-Net: best epoch 360; (**C**) R2U-Net: best epoch 270; and (**D**) ResU-Net: best epoch 950. Green dashed vertical line = best epoch (minimum validation loss). *Y*-axis range 0–0.16 unified across all panels.

**Figure 3:**
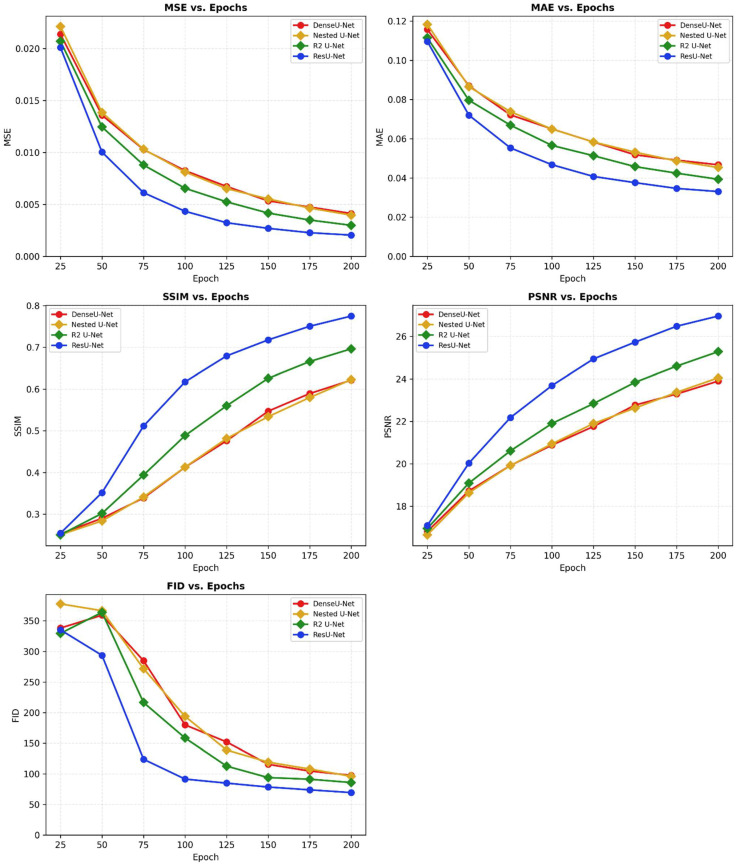
Test-set performance metrics across training epochs (epochs 25–200). Changes in MSE, MAE, SSIM, PSNR, and FID on the test set at 25-epoch intervals. Colour coding: Dense U-Net = red, Nested U-Net = yellow, R2U-Net = green, and ResU-Net = blue. ResU-Net demonstrated the fastest convergence and the best performance at epoch 200.

To analyze the learning progress of all models, performance was measured at 25-epoch intervals (Figure 2). Each model was trained up to 1000 epochs with early stopping (patience = 50 epochs for all models), and changes in the five metrics were tracked at 25-epoch intervals over the full training duration (Figure 3). Convergence epochs were: Dense U-Net 285, Nested U-Net 360, R2U-Net 270, and ResU-Net 950. The following characteristic patterns were observed:

ResU-Net: Showed the fastest convergence rate among all models. SSIM rapidly improved to 0.6113 at 75 epochs, and by 125 epochs, it had already reached excellent performance with SSIM of 0.7879 and PSNR of 25.56 dB. Performance continued to improve steadily reaching best performance at epoch 950.

R2U-Net: Showed relatively slow convergence in the early stages (25–75 epochs), but performance significantly improved after 100 epochs. SSIM improved dramatically from 0.6539 to 0.7124 between 125 and 150 epochs.

Nested U-Net: Showed learning patterns similar to R2U-Net overall, but variability was observed particularly in FID scores. FID temporarily increased from 165.39 to 176.62 between epochs 50 and 75 before decreasing again.

Dense U-Net: Showed the slowest convergence rate among all models. Even at 200 epochs, it only reached performance levels equivalent to 100–125 epochs of other models. Performance improvement was very limited during the initial 50 epochs.

**Figure 4:**
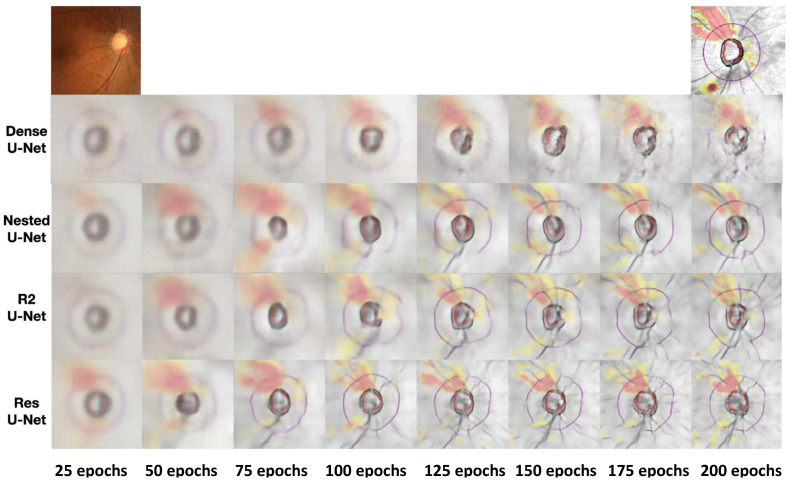
Qualitative comparison of generated RNFL thickness maps. RNFL thickness maps generated by the four architectures at 25-epoch intervals alongside the reference fundus photograph (**top left**) and actual OCT-derived RNFL map (**top right**). The warmer the color is, the thinner the relative thickness is. The inner circle denotes the optic disc boundary, while the outer circle indicates the peripapillary region of interest. A representative case from the test set was selected (intermediate glaucoma severity, good image quality). Quantitative performance across all 500 test-set eyes is reported in Table 2.

Figure 4 shows the quality changes in generated images in each architecture. A representative case from the held-out test set was selected to illustrate learning progression; this case had intermediate glaucoma severity and good image quality. Note that this single case may not reflect the full range of performance variability across cases with different disease severity, image quality, or structural damage patterns.

ResU-Net began capturing the basic structure of RNFL even at the initial 25 epochs and successfully started reproducing the main structural features of RNFL by 75 epochs. By 125 epochs, it could generate images quite similar to real OCT, and at 200 epochs, it produced high-quality images accurately reproducing even fine details.

R2U-Net generated blurry images lacking details at initial 25–50 epochs but showed dramatic quality improvement after 100 epochs.

Nested U-Net generated quite blurry images until 75 epochs, and the basic structure of RNFL began to become distinct from 100 epochs.

Dense U-Net showed the slowest quality improvement. In early epochs (25–75), the model generates blurry images with poor structural definition.

Mean inference time per image for all four architectures is reported in Table 2, measured on NVIDIA H100 80 GB × 2 GPU (Google Colab Enterprise, Vertex AI). All models demonstrated inference times below 200 ms per image, confirming compatibility with practical clinical deployment. ResU-Net achieved an inference time of 100.7 ± 1.1 ms, supporting its feasibility for both real-time and batch-processing workflows.

## 8. Discussion

This study’s findings demonstrate significant implications for deep learning applications in ophthalmology. Our comprehensive comparison of four U-Net variants reveals important insights about architectural design choices for medical image transformation tasks.

The superior performance of ResU-Net across all metrics highlights a critical principle in medical AI development: architectural elegance often trumps complexity. While more complex architectures like Dense U-Net offer theoretical advantages in information flow, ResU-Net’s successful balance between depth and structural simplicity proved optimal for this specific task. The efficient integration of residual connections within the U-Net architecture addresses the vanishing gradient problem without introducing excessive parameters, creating an ideal foundation for the delicate task of RNFL reconstruction from fundus images.

The varying learning patterns observed across architectures reveal important considerations for model selection in medical imaging. ResU-Net’s rapid convergence suggests it efficiently captures the fundamental relationship between fundus features and RNFL structure, suggesting it may offer practical advantages for future clinical evaluation once prospective validation has been completed. In contrast, the delayed convergence patterns of more complex architectures like R2U-Net and Dense U-Net suggest these models may require significantly more computational resources and training time without proportional performance gains.

R2U-Net’s performance trajectory, with dramatic improvement after 100 epochs, suggests its recursive structure eventually develops powerful feature representations, though at the cost of extended training time. This trade-off between training duration and final performance must be carefully evaluated in practical implementation contexts, especially in resource-limited settings.

The poor performance of Dense U-Net, despite its theoretical advantages in feature reuse, serves as a cautionary example against automatically applying architectures successful in general computer vision to specialized medical tasks.

These findings emphasize the importance of task-specific architecture selection in medical imaging. The performance discrepancies observed challenge the assumption of cross-task architecture transferability, even within the same domain of ophthalmology. This reinforces the need for comparative studies when developing AI solutions for specific clinical applications.

From a clinical perspective, the substantially improved performance metrics achieved by ResU-Net (SSIM = 0.9163, PSNR = 32.19 dB) after extended training represent a potential contribution toward improving accessible glaucoma assessment in resource-limited settings, though prospective clinical validation, external generalisability testing, and regulatory evaluation are required before clinical deployment is considered [8,28]. The ability to extract OCT-like structural information from widely available fundus photographs represents a significant step toward more accessible glaucoma screening and monitoring in underserved regions.

Furthermore, our image-to-image transformation approach offers advantages beyond simple numerical predictions or binary classifications. By generating complete RNFL thickness maps that visualize the entire distribution pattern, clinicians gain richer diagnostic information than methods that produce only averaged thickness values or classification outcomes. This spatial information could potentially improve detection of localized defects and subtle progression patterns critical for glaucoma management.

Future developments should explore integrating additional clinical data modalities. Incorporating visual field results, intraocular pressure measurements, or demographic risk factors could further enhance the model’s diagnostic capabilities. Future work should also explore alternative generative architectures. While the present study focused on U-Net variants selected for their established efficacy in biomedical image segmentation and transformation tasks [26], other generative frameworks—including generative adversarial networks (GANs) and hybrid architectures combining elements of multiple U-Net variants—may offer further improvements in perceptual quality, as reflected in FID scores [21]. The style-transfer approach of Chen et al. [10] represents one such alternative, and direct architectural comparisons in future studies would clarify the relative advantages of each generative strategy for this specific task.

This study has distinct differences from existing approaches to glaucoma diagnosis using fundus photographs. Most recent deep learning-based studies have focused on regression or classification models that either predict average RNFL thickness or classify the presence of glaucoma from fundus photographs [8,14,28]. These approaches provide clinically useful information but have fundamental limitations in the depth and range of information obtainable from fundus photographs.

In contrast, the U-Net-based image transformation model proposed in this study goes beyond simple measurement prediction or binary classification by directly generating complete RNFL thickness maps from fundus photographs, providing detailed structural information similar to OCT scans. This represents significant innovation: enhanced visual interpretability—while regression models provide single numerical values or limited sectoral measurements, our approach visualizes the RNFL thickness distribution across the entire area surrounding the optic nerve, providing information that clinicians can interpret more intuitively [13,14]. The generated RNFL thickness maps allow for assessment beyond simply determining the presence of glaucoma, enabling evaluation of glaucoma progression, damage patterns, and even the location of localized defects. This complements the progression detection approach over time proposed by Medeiros et al. [15] while providing more detailed information on structural changes.

Previous research has demonstrated the generalizability of fundus photograph-based glaucoma detection [8,28,29,30]. This study goes a step further by presenting the possibility of obtaining detailed RNFL information similar to OCT without expensive OCT equipment.

This work represents a potential contribution toward improving accessible glaucoma assessment in resource-limited settings [8,28], contingent upon prospective clinical validation and formal regulatory assessment. No claims regarding paradigm change or clinical equivalence to OCT are made in this study.

The clinical deployment of AI-generated diagnostic images raises important ethical and regulatory considerations. Any AI system intended for clinical decision support must undergo formal regulatory evaluation—such as the FDA 510 (k) or De Novo pathway, or EU MDR conformity assessment—and demonstrate safety and efficacy in prospective clinical trials before clinical use [8,14]. Questions of clinical liability in cases of AI-assisted diagnostic error remain unresolved, and transparency regarding the synthetic nature of AI-generated RNFL maps must be maintained in any implementation context. Additionally, the risk of algorithmic bias in patient populations underrepresented in the training dataset requires careful evaluation prior to deployment in diverse clinical settings.

Several limitations of this study must be acknowledged. First, the generated RNFL maps show residual differences from actual OCT-based measurements; the achieved SSIM = 0.9163 and PSNR = 32.19 dB for ResU-Net indicate high but not clinically validated image-level similarity, and formal equivalence to OCT measurements has not been demonstrated. Second, this is a retrospective, single-centre study; generalisability to other OCT devices, imaging protocols, institutions, or patient populations—including different ethnicities, glaucoma subtypes, and optic disc morphologies—has not been established [8,28]. Third, model evaluation was conducted exclusively using image similarity metrics; clinical validation against diagnostic outcomes such as glaucoma detection accuracy, sector-level RNFL thickness agreement, or visual field correlation has not been performed. A formal qualitative assessment by ophthalmologists comparing predicted and actual RNFL maps—using a standardized grading instrument and appropriately blinded readers—is identified as the primary goal of planned follow-up studies. Fourth, the dataset comprised exclusively glaucoma patients; model performance on healthy eyes, ocular hypertension, or other optic neuropathies is unknown. Fifth, all results are based on a single training run per architecture; variability across independent training runs (mean ± SD) was not assessed due to computational resource constraints. Sixth, subgroup analyses stratified by disease severity, image quality grade, or axial length were not performed; performance in subgroups with advanced glaucoma, poor image quality, or media opacity may differ from overall results. Seventh, the ethical, regulatory, and liability frameworks governing the clinical deployment of AI-generated diagnostic images remain undefined; regulatory clearance and prospective trials are required before any clinical use.

## 9. Conclusions

In conclusion, we confirmed that ResU-Net consistently outperformed other architectures across all performance metrics, achieving SSIM = 0.9163 and PSNR = 32.19 dB after extended training—a 108% and 67% improvement, respectively, over the best published benchmark for this task. Through this comparative study of U-Net-based deep learning models that generate RNFL thickness maps directly from fundus photographs, we demonstrated the technical feasibility of this approach as a potential supplement to OCT in accessible glaucoma assessment programmes. These findings provide a technical foundation for future prospective validation, external generalisability testing, and regulatory evaluation required before clinical deployment.

## Figures and Tables

**Table 1:** Baseline demographic and clinical characteristics (N = 5000 patients).

Characteristics		
**Diagnosis (%)**	NTG	2990 (59.8%)
	POAG	1875 (37.5%)
	PACG	135 (2.7%)
**Mean age (year)**		61.49 ± 15.74
**Male gender (%)**		2345 (46.9%)
**MD (dB)**		−4.90 ± 5.95
**PSD (dB)**		4.62 ± 3.90
**VFI (%)**		86.95 ± 18.50
**IOP (mmHg)**		13.57 ± 3.11

Characteristics are summarized as mean ± SD or n (%). Glaucoma type: normal-tension glaucoma n (%), primary open-angle glaucoma n (%), primary angle-closure glaucoma n (%); age: [mean ± SD years]; gender (male): n (%); mean deviation (MD): [mean ± SD dB]; pattern standard deviation (PSD): [mean ± SD dB]; visual field index (VFI): [mean ± SD %]; intraocular pressure (IOP): [mean ± SD mmHg].

**Table 2:** Performance on the test set at the best-performing epoch for the four architectures.

Model	MSE	MAE	SSIM	PSNR (dB)	FID	Inference Time † (ms)
Dense U-Net	0.00380	0.04447	0.6621	24.42	62.22	155.4 ± 1.3
Nested U-Net	0.00193	0.03247	0.7671	27.19	76.18	77.2 ± 1.1
R2U-Net	0.00229	0.03492	0.7475	26.45	81.96	85.1 ± 1.0
**ResU-Net**	**0.00061**	**0.01877**	**0.9163**	**32.19**	**30.08**	**100.7 ± 1.1**

Bold row *=* best-performing model across all metrics. † Mean ± SD inference time per image measured on NVIDIA H100 80 GB × 2 GPU (Google Colab Enterprise, Vertex AI, a3-highgpu-2 g instance). Values averaged over 100 consecutive inference runs following 10 warm-up runs. Abbreviations: MSE = Mean Squared Error; MAE = Mean Absolute Error; SSIM = Structural Similarity Index Measure; PSNR = Peak Signal-to-Noise Ratio; FID = Fréchet Inception Distance. Training configuration: All models trained with Adam optimiser (lr = 2 × 10^−4^, batch = 4, MSE loss). Maximum 1000 epochs; early stopping patience = 50 epochs for all models. Best model selected by minimum validation loss. ResU-Net reached best performance at epoch 950 with early stopping triggered at epoch 1000.

## Data Availability

The underlying training/validation datasets for this study is not publicly available but may be made available to qualified researchers on reasonable request from the corresponding author.

## Supplementary Materials

The following supporting information can be downloaded at https://www.mdpi.com/article/10.3390/life16040559/s1, S1: Detailed Description of Performance Evaluation Metrics; Table S1: Comparison with Related Studies; Table S2: Metric Selection Justification Summary.
