# Supplementary material for: Deep Learning-Based Generation of Retinal Nerve Fibre Layer Thickness Maps from Fundus Photographs: A Comparative Analysis of U-Net Architectures for Accessible Glaucoma Assessment
Source: Life (Basel). 2026 Mar 29;16(4):559. doi: 10.3390/life16040559 (PMC13117065; doi:10.3390/life16040559)
Supplement: Supplementary file 1 [file life-16-00559-s001.zip › life-4195689-supplementary.pdf]

Supplementary Materials

S1 — Metric rationale (5 subsections)

S2 — Related studies comparison (Table S1)

S3 — Metric summary (Table S2)

S1. Detailed Description of Performance Evaluation Metrics

In this study, five quantitative metrics were used to evaluate the quality of the generated retinal nerve fiber layer (RNFL) thickness maps. Each metric captures a different aspect of image similarity between the predicted and actual OCT-derived RNFL maps. The rationale for selecting each metric and its clinical relevance to RNFL assessment are described below. All models were trained for up to 1,000 epochs with early stopping (patience = 50 epochs, based on validation loss) and an Adam optimizer (learning rate = 2×10^{-4}, batch size = 4, MSE loss function).

S1.1. Mean Squared Error (MSE)

MSE quantifies the average squared pixel-wise difference between the predicted and actual RNFL thickness maps:[19]

$$MSE = (1/N) \times \Sigma (x_i - y_i)^2$$

where x_i and y_i represent pixel values of the original and predicted images respectively, and N is the total number of pixels.

Rationale for selection: MSE directly reflects pixel-level deviation in RNFL thickness values. Because the generated maps encode thickness information through pixel intensity, MSE effectively measures how closely the predicted values approximate the true OCT measurements at each spatial location. Lower MSE values indicate smaller prediction errors across the entire RNFL map.

Clinical relevance: In clinical practice, RNFL thickness measurements are used to detect thinning associated with glaucomatous damage. MSE provides a global measure of prediction accuracy; clinically meaningful RNFL thickness differences (typically >5–10 μm) would be reflected as elevated MSE values. However, MSE treats all spatial locations equally and may not adequately capture localised defects that are clinically significant.

S1.2. Mean Absolute Error (MAE)

MAE measures the average absolute pixel-wise difference between predicted and actual values:[20]

$$MAE = (1/N) \times \Sigma |x_i - y_i|$$

Rationale for selection: Unlike MSE, MAE is less sensitive to large outlier errors and provides a linear measure of average prediction deviation. This property is particularly relevant for RNFL map evaluation, where a few pixels with large errors (e.g., at boundary regions) should not disproportionately dominate the overall assessment. MAE offers a more robust measure of typical prediction accuracy across the map.

Clinical relevance: MAE can be interpreted as the average magnitude of thickness prediction error per pixel. When pixel intensity is calibrated to RNFL thickness values, MAE provides a clinically intuitive measure of how far, on average, the predicted thickness deviates from the actual OCT measurement. This complements MSE by reflecting typical rather than squared error magnitude.

S1.3. Structural Similarity Index Measure (SSIM)

SSIM evaluates structural similarity between two images by comparing luminance, contrast, and structural patterns:[17]

$$SSIM(x,y) = (2\mu_x\mu_Y + C_1)(2\sigma_{xY} + C_2) / (\mu_x^2 + \mu_Y^2 + C_1)(\sigma_x^2 + \sigma_Y^2 + C_2)$$

where μ_x, μ_Y = mean luminance; σ_x^2, σ_Y^2 = variance (contrast); σ_{xY} = covariance (structural similarity); C_1, C_2 = stabilising constants. SSIM ranges from 0 to 1, with higher values indicating greater similarity.

Rationale for selection: SSIM is specifically designed to assess perceptual image quality by evaluating structural patterns rather than simple pixel differences. For RNFL thickness maps, the spatial distribution pattern — including the characteristic double-hump profile of RNFL thickness around the optic disc — is clinically more informative than individual pixel values. SSIM captures whether these structural patterns are preserved in the generated maps.

Clinical relevance: RNFL assessment in glaucoma relies heavily on recognising spatial patterns of thickness distribution. Clinicians evaluate the superior and inferior arcuate bundles, identify focal thinning, and assess the overall symmetry of the RNFL profile. SSIM directly measures the preservation of such spatial structures. Chen et al. reported SSIM = 0.44 for a similar fundus-to-RNFL map generation task2; the present study achieved **SSIM = 0.9163** with ResU-Net following retraining to convergence (best epoch: 950 of 1,000, early stopping patience = 50), representing a 108% improvement in structural fidelity over that benchmark.

S1.4. Peak Signal-to-Noise Ratio (PSNR)

PSNR measures the ratio between the maximum possible signal power and reconstruction error, expressed in decibels (dB):[18]

$$PSNR = 10 \times \log_{10} (MAX^2 / MSE)$$

where MAX is the maximum possible pixel value in the image (e.g., 255 for an 8-bit image).

Rationale for selection: PSNR provides a standardised measure of overall image reconstruction quality on a logarithmic scale, making it sensitive to both global and local differences. It is one of the most widely used metrics in image generation and reconstruction studies, facilitating direct comparison across different studies and imaging modalities.

Clinical relevance: Higher PSNR values indicate that the generated RNFL map contains less reconstruction noise relative to the actual OCT map, meaning the predicted thickness information is more signal-faithful. Chen et al.[10] reported PSNR = 19.31 dB; the present study achieved **PSNR = 32.19 dB** with ResU-Net, exceeding 30 dB and representing a 67% improvement in signal quality. While this level of PSNR suggests high reconstruction fidelity, clinical decision-making should continue to rely on actual OCT measurements until prospective validation is completed.

S1.5. Fréchet Inception Distance (FID)

FID measures the distance between two image distributions in a feature space extracted by the Inception v3 network:[21]

$$FID = ||\mu_r - \mu_g||^2 + Tr(\Sigma_r + \Sigma_g - 2(\Sigma_r\Sigma_g)^{(1/2)})$$

where μ_r, μ_g = mean feature vectors; Σ_r, Σ_g = covariance matrices of the real and generated image distributions; Tr = matrix trace.

Rationale for selection: Unlike pixel-level metrics (MSE, MAE, PSNR) and structural metrics (SSIM), FID evaluates overall distributional similarity between generated and real images at a higher semantic level. This is particularly important for image generation tasks, as two images can have similar pixel-level metrics but different perceptual quality. FID captures whether the generated RNFL maps, as a population, exhibit the same statistical characteristics as real OCT-derived maps.

Clinical relevance: FID assesses whether the collection of generated RNFL maps exhibits realistic variation patterns consistent with the actual patient population. A low FID score indicates that the generated maps collectively reproduce the natural variability of RNFL patterns seen in clinical OCT scans — important for ensuring the model does not produce stereotyped outputs that fail to represent the diversity of glaucomatous damage patterns encountered in clinical practice.

S2. Comparison with Related Studies (Table S1)

Table S1 presents a comparison of the performance metrics reported in the present study with those from related studies performing fundus photograph-based RNFL assessment. Values reflect the final retrained ResU-Net results (1,000-epoch limit, early stopping patience = 50, best epoch: 950).

Table S1. Comparison of performance metrics with related studies.

Study	Task	Model	SSIM	PSNR (dB)	Data Size	Approach
Chen et al. (2022) [10]	Fundus → RNFL map	U-Net + style transfer	0.44	19.31	1,120 eyes	Style transfer
Medeiros et al. (2019) [14]	Fundus → RNFL thickness	CNN	N/A	N/A	32,820 pairs	Regression (r = 0.832)
Yang et al. (2022) [9]	Fundus → RNFL thickness	CNN	N/A	N/A	310 eyes	Regression (Se 92%, Sp 86.9%)
Xu et al. (2024) [13]	Fundus → RNFL thickness	CNN	N/A	N/A	Not specified	Regression
**Present study (ResU-Net)**	**Fundus → RNFL map**	**ResU-Net**	**0.9163**	**32.19**	**5,000 pairs**	**Image-to-image (direct)**

N/A = not applicable (regression-based studies report MAE in μm rather than image-level SSIM/PSNR). Se = sensitivity; Sp = specificity.

Chen et al.[10] performed the most directly comparable study, generating RNFL thickness distribution maps from fundus photographs using a style-transfer approach. Their reported SSIM = 0.44 and PSNR = 19.31 dB provide the primary benchmark for the present study. The substantial improvement achieved here (SSIM = 0.9163, PSNR = 32.19 dB) may be attributed to: (1) the larger training dataset (5,000 pairs vs. 1,120 eyes); (2) the use of specialised U-Net variants with architectural innovations, particularly residual connections in ResU-Net;[26],[27] and (3) extended training to full convergence (best epoch: 950, up to 1,000-epoch limit with early stopping patience = 50).

Medeiros et al.[14], Yang et al.[9], and Xu et al.[13] employed regression-based approaches that predict numerical RNFL thickness values rather than generating spatial maps. While these approaches reported strong discriminative performance, they inherently lose spatial distribution information. The image-to-image transformation approach in the present study preserves the full two-dimensional topographic structure of the RNFL, which may provide clinicians with more comprehensive information regarding the location, extent, and pattern of RNFL thinning — particularly relevant for identifying sector-specific defects and monitoring glaucoma progression.[15]

S3. Metric Selection Justification Summary (Table S2)

The five metrics were selected to provide a comprehensive, multi-level evaluation of the generated RNFL thickness maps across three complementary dimensions: pixel-level accuracy (MSE, MAE), structural pattern fidelity (SSIM, PSNR), and population-level distributional realism (FID).

Table S2. Summary of metric selection rationale and limitations.

Metric	What it measures	Clinical relevance to RNFL	Advantage	Limitation
MSE[11]	Average squared pixel error	Reflects overall thickness prediction accuracy	Directly penalises large errors; sensitive to gross discrepancies	Sensitive to outliers; ignores spatial structure
MAE[12]	Average absolute pixel error	Intuitive measure of typical pixel-level thickness deviation	Robust to outlier pixels; linear and easy to interpret	Does not capture spatial or structural patterns
SSIM[13]	Structural pattern similarity	Preservation of arcuate bundle morphology and focal RNFL defects	Perceptually motivated; aligns with clinical pattern recognition	May not detect subtle localised defects
PSNR[14]	Signal-to-noise ratio (dB)	Overall reconstruction quality; enables direct cross-study comparison	Widely used; logarithmic scale sensitive to fine pixel-level differences	Less intuitive for direct clinical interpretation
FID[15]	Distribution-level similarity	Ensures generated maps reproduce realistic population-level RNFL variability	Captures perceptual realism beyond individual pixel metrics	Requires sufficient sample size; depends on Inception v3 network features

Together, MSE and MAE evaluate pixel-level accuracy; SSIM and PSNR evaluate structural pattern fidelity; and FID evaluates population-level distributional realism. This multi-level framework provides a more comprehensive assessment than any single metric alone and was specifically selected to align with both the technical requirements of image generation evaluation and the clinical requirements of RNFL map assessment in glaucoma.

Note on References

All superscript reference numbers in this file correspond directly to the numbered references in the main manuscript (Refs. 1–30). No additional references beyond those in the main manuscript have been cited in this Supplementary Materials file.
